# Exceptional Renal Metastasis from Adenoid Cystic Carcinoma of the Nasal Cavity and Literature Review

**DOI:** 10.15586/jkcvhl.v8i3.173

**Published:** 2021-09-23

**Authors:** Jihene Feki, Maissa Lajnef, Manel Mallouli, Kheireddine Ben mahfoudh, Tahia Boudawara, Afef Khanfir

**Affiliations:** 1 Department of Medical Oncology, Habib Bourguiba Hospital, University of Sfax, Sfax, Tunisia;; 2 Department of Pathology, Habib Bourguiba Hospital, University of Sfax, Sfax, Tunisia;; 3 Department of Radiology, Habib Bourguiba Hospital, University of Sfax, Sfax, Tunisia

**Keywords:** adenoid cystic, carcinoma, nasal cavity, kidney, renal metastases

## Abstract

Adenoid cystic carcinoma (ACC) is a rare malignant cancer that arises from secretory glands. Slow growth, perineural invasion, and late recurrences are the main characteristics of ACC. Only few cases of kidney metastases from ACC have been reported in the literature. We report here the case of a 66-year-old female patient who presented with bilateral renal metastases from ACC of the nasal cavity, detected 14 years after treatment of primary tumor and 6 years after metastasectomy of lung metastases. Histological examination confirmed diagnosis and the patient was treated with systemic chemotherapy. Radiological evaluation showed stability of the disease. However, a progression with occurrence of metastases in other sites (lung and bones) has been observed after 7 months. She is still receiving second-line chemotherapy. To the best of our knowledge, this is the second case of kidney metastases from ACC of the nasal cavity.

## Introduction

Adenoid cystic carcinoma (ACC) is an uncommon neoplasm accounting for approximately 1% of all head and neck cancers (1). It is characterized by indolent clinical course and a high incidence of late metastases. We describe herein an uncommon case of renal metastases from ACC of the nasal cavity. This case improved our knowledge about this extremely rare site of metastases from ACC and emphasized the importance of a long-term follow up to detect any late metastases.

## Case Report

**Figure 1:**
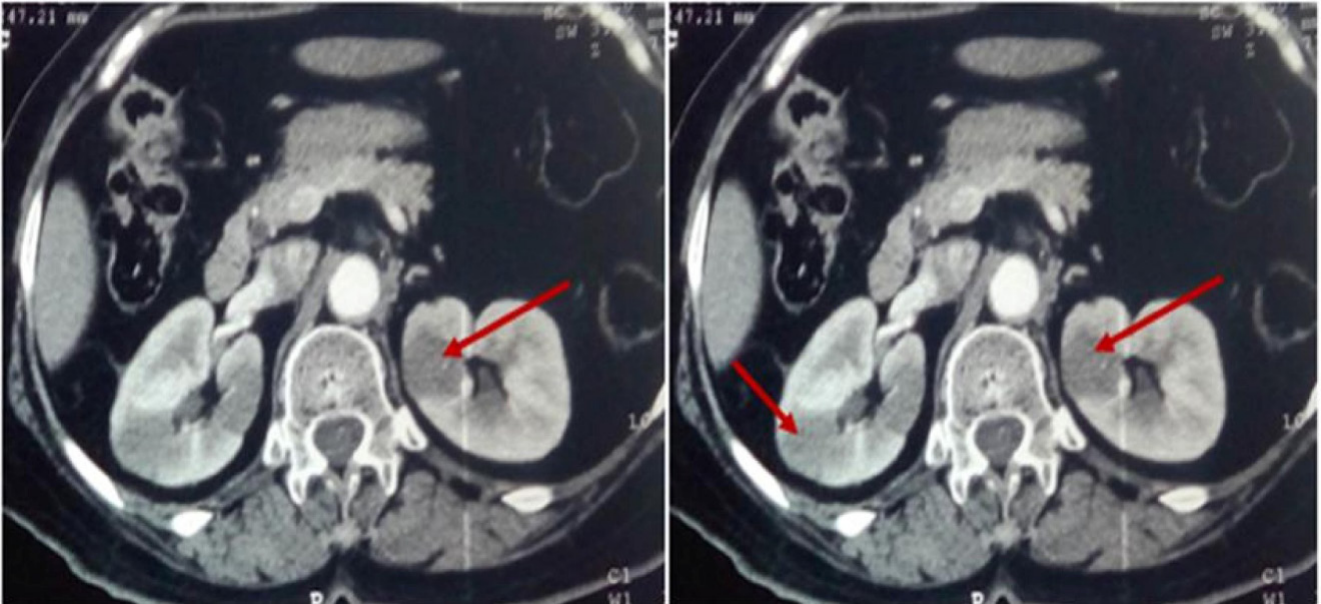
CT scan showing bilateral renal lesions.

In 2005, a 66-year-old female consulted for nasal obstruction and rhinorrhea evolving since 8 months. Clinical examination documented a left polypoid nasal lesion; tumor biopsy revealed an ACC. The patient underwent surgery followed by radiation therapy (64Gy). During follow-up, after 8 years, the patient presented a distant recurrence of the disease with solitaire lung metastases. No local relapse was observed. Metastasectomy of the lesion was practiced with economic margins, followed by radiotherapy (60Gy). In April 2019, the patient presented at the hospital for a pain in the right flank. No other symptoms were reported. Computed tomography (CT) of the abdomen showed multiple hypodense nodular lesions of the two kidneys, the larger one measuring 30 × 25 × 40 mm in the right kidney (Figure 1).

**Figure 2:**
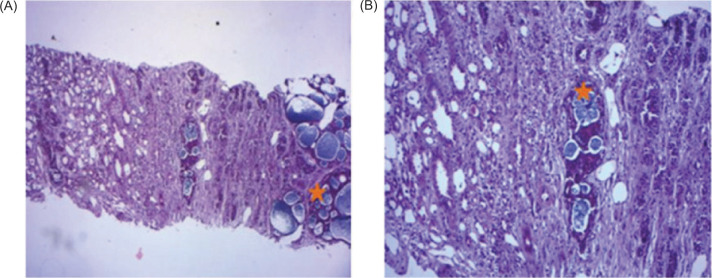
(A) H&E photomicrographs showing renal localization of adenoid cystic carcinoma (*) with various growth patterns: Cribriform and trabecular, a characteristic cribriform pattern of adenoid cystic carcinoma (hematoxylin-eosin, ×100). (B) Cribriform growth pattern displaying several prominent pseudocysts surrounded by basaloid cells (hematoxylin-eosin, ×200); Arrows indicate renal tubules.

The patient was referred fora CT-guided renal biopsy. Histology showed a cribriform growth pattern with two types of cells, monomorphic epithelial cells with a basophilic cytoplasm and myoepithelial cells, which was consistent with kidney metastases from ACC (Figure 2).

The patient was treated with chemotherapy: CAP (Cyclophosphamide-Adriamycin-Cisplatinum) regimen (cyclophosphamide 500 mg/m^2^, adriamycin 50 mg/m^2^, and cis-platinum 50 mg/m^2^). The radiological evaluation by a CT scan after six cycles of chemotherapy showed a stable disease. Within 7 months, a progression of the disease was observed with the occurrence of lung and bone metastases. Therefore, second-line chemotherapy was indicated: capecitabine with bisphosphonates. Actually, the patient is still alive. After four cycles of capecitabine, a further line of chemotherapy has been started because of a progression of bone metastases. She is still receiving chemotherapy based on paclitaxel with disease stability.

## Discussion

ACC is a rare malignancy, accounting for 1% of all head and neck tumors and 10% of malignant salivary glandneoplasm (1). Most frequently, ACC involves minor salivary glands. The involvement of the nasal cavity is less common. ACC is characterized by low growth, perineural invasion, and a high frequency of late local recurrences and hematogenous metastases (2). The lung is the most common site of metastases, followed by bones, liver, and occasionally the brain (3). Renal metastases of ACC are extremely rare, and only few cases have been reported in the literature. Among these reported cases, metastases to the kidney originated from different sites such as breast, lung, auditory canal, and lacrymal gland. However, to our knowledge, this is the second case describing renal metastasis from the nasal cavity. In our case, the patient experienced two relapses: at first in the lung after 8 years of treatment of the primary tumor and then in the kidney after a period of 6 years. This confirms the high tendency of ACC to metastasize. Renal metastases are usually asymptomatic and due to their location in the vascular plexus cortex, hematuria is rarely observed (4). In the current case, urinary symptoms have not been reported but a pain in the right flank was observed. Several CT features of renal metastases have been reported such as solid, cystic masses or hemorrhagiclesions. Pet/CT findings may be helpful in the detection of those lesions and to differentiate between benign and malignant lesions. Qiu et al. reported a case of a singular metastatic ACC of the right kidney 3 years after the treatment of an ACC of submandibular gland. PET-CT revealed a moderate FDG intake by the renal lesion, and diagnosis was confirmed by histological examination after a right radical nephrectomy (4). Histological diagnosis of renal metastases remains necessary to avoid misdiagnosing primary renal tumors. Management of kidney metastases of ACC is not well defined. Up to now, surgery is the main treatment method proposed (5). However, this approach wasn’t possible in our case because of the multiplicity of the lesions. Other treatment modalities, such as thermoablation, renal embolization, may be proposed in some patients, especially with painful lesions (6). Systemic chemotherapy with agents, such as cyclophosphamide, cis-platinum, 5 fluouracil, and doxorubin, may have some efficacy against metastatic ACC and must be considered when locoregional treatment is not indicated. Our patient presented a stabilization of the disease with CAP regimen; however, a progression with the occurrence of metastases in other sites was observed.

## Conclusion

The particularity of this case relies on the rarity of renal metastasis from ACC, specifically of nasal cavity, and the complexity of its diagnosis and management. Likewise, clinicians must be aware of the existence of this condition anda careful monitoring is recommended to detect any additional metastases from ACC.
